# Molecular Response to Nanopatterned Implants in the Human Jaw Bone

**DOI:** 10.1021/acsbiomaterials.1c00861

**Published:** 2021-12-01

**Authors:** Dimitrios Karazisis, Omar Omar, Sarunas Petronis, Peter Thomsen, Lars Rasmusson

**Affiliations:** † Department of Biomaterials, Institute of Clinical Sciences, Sahlgrenska Academy, University of Gothenburg, 405 30 Gothenburg, Sweden; ‡ Department of Oral and Maxillofacial Surgery, Sahlgrenska Academy, University of Gothenburg, 405 30 Gothenburg, Sweden; § Department of Biomedical Dental Sciences, College of Dentistry, Imam Abdulrahman bin Faisal University, Dammam 34212, Saudi Arabia; ∥ Chemistry, Biomaterials and Textiles, RISE Research Institutes of Sweden, 501 15 Borås, Sweden; ⊥ Maxillofacial Unit, Linköping University Hospital, 581 85 Linköping, Sweden

**Keywords:** osseointegration, nanomedicine, nanotopography, gene expression, osteogenic activities, human jaw

## Abstract

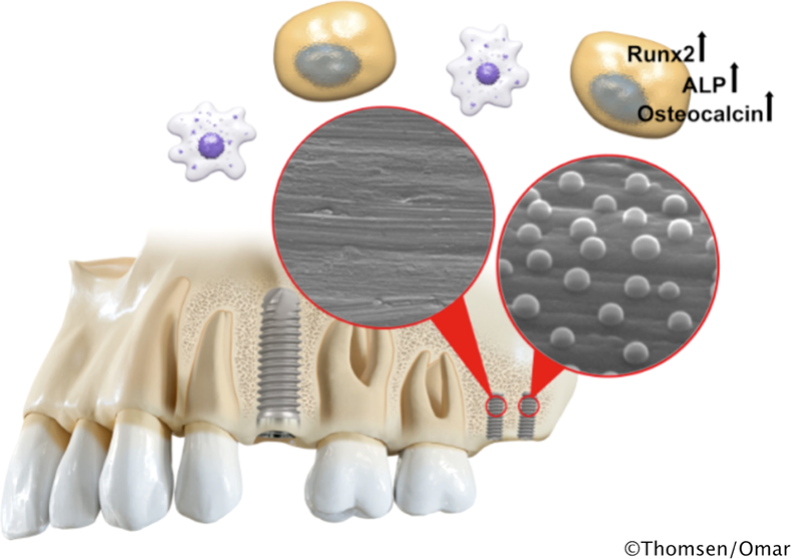

Implant surface modification by nanopatterning is an interesting route for enhancing osseointegration in humans. Herein, the molecular response to an intentional, controlled nanotopography pattern superimposed on screw-shaped titanium implants is investigated in human bone. When clinical implants are installed, additional two mini-implants, one with a machined surface (M) and one with a machined surface superimposed with a hemispherical nanopattern (MN), are installed in the posterior maxilla. In the second-stage surgery, after 6–8 weeks, the mini-implants are retrieved by unscrewing, and the implant-adherent cells are subjected to gene expression analysis using quantitative polymerase chain reaction (qPCR). Compared to those adherent to the machined (M) implants, the cells adherent to the nanopatterned (MN) implants demonstrate significant upregulation (1.8- to 2-fold) of bone-related genes (RUNX2, ALP, and OC). No significant differences are observed in the expression of the analyzed inflammatory and remodeling genes. Correlation analysis reveals that older patient age is associated with increased expression of proinflammatory cytokines (TNF-α and MCP-1) on the machined implants and decreased expression of pro-osteogenic factor (BMP-2) on the nanopatterned implants. Controlled nanotopography, in the form of hemispherical 60 nm protrusions, promotes gene expressions related to early osteogenic differentiation and osteoblastic activity in implant-adherent cells in the human jaw bone.

## 1 Introduction

The implantation of materials in bone has revolutionized their application in orthopedic and cranio-maxillo-facial contexts. The science underpinning the long-term survival and success rates in the range of 80–99% for oral implants,^1−5^ hip and knee arthroplasties,^6,7^ amputation prostheses,^8−10^ and bone-anchored hearing devices^11−14^ goes back to the concept of osseointegration. This concept includes not only structural adaptation and/or bonding of organic and inorganic components but also the preceding cellular and molecular processes.^15−18^ The latter processes occur in a narrow interface zone between the surface of the material and tissue.^17,19^ Surface modifications have been an essential method by which material properties are optimized for oral implants.^20^ The majority of these modifications entail subtractive techniques as blasting alone, blasting and etching or anodization in combinations.^21^ Generally, the resulting range of topographic features on multiple scales hinders the detailed understanding of the role of specific length scale surface properties in cellular, structural, biomechanical, and clinical outcomes. Strategies to enable the elucidation of such correlations on the nanoscale level include nanoprocessing techniques, allowing the experiments to be reproducible and consistent.^22^

The methods applied to produce nanopatterns can be categorized as either subtractive or additive. Acid etching is the most common subtractive method used for creating random nanotopographies on implants.^23,24^ Lithographic techniques, using electron beam,^25^ X-ray,^26^ or laser^27^ as the source of radiation, result in ordered nanopatterns, commonly applied on smooth and flat composite materials and wafers for *in vitro* studies. Additive nanofabrication methods, on the other hand, are commonly based on low-voltage anodization process leading to partially ordered nanotube formation.^28,29^ In this context, the authors have emphasized the advantage of electrochemical anodization techniques for the fabrication of controlled nanopatterns while preserving microscale features of the underlying implant.^30,31^ Other additive techniques, such as colloidal lithography,^32,33^ nanoimprinting, and replica molding,^22^ provide possibilities to fabricate controlled nanopatterns even on complex three-dimensional (3D) implants.

Rapidly emerging research on the role of nanoscale features in osseointegration has shown that nanotopography promotes mesenchymal stem cell (MSC) adhesion, proliferation, migration, and osteogenic differentiation *in vitro*.^25,29,34,35^ Furthermore, enhanced *in vitro* osteogenic activity over the short term and matrix mineralization over the long term have been confirmed.^23,24,27,36,37^ In addition, nanotopography has been suggested to exert immunomodulatory effects via decreased adhesion, proliferation, and/or proinflammatory cytokine expression of macrophages.^38−40^

A major question is whether nanoscale topography influences specific processes in the *in vivo* microenvironment. Hitherto, studies using different animal models suggest that nanotopography enhances bone formation, as determined by increased bone-implant contact (BIC),^29,37,41^ and increases implant stability, as judged by biomechanical testing.^42−44^ Furthermore, gene expression analyses at the bone-implant interface have shown enhanced osteogenic activity and/or downregulation of proinflammatory cytokines.^28,32,33^ Although the mechanism underlying nanotopographic mediated modulation of molecular activities has not yet been detailed, it is evident that the inflammatory, anabolic and catabolic processes adjacent to nanopatterned surfaces differ from those at other length scales. Moreover, since these processes are intimately linked with structural and biomechanical parameters,^44^ there is ample opportunity for human studies and clinical translation.

To the best of the authors’ knowledge, no data on the role of ordered nanoscale properties on implants intended for bone anchoring in humans are available. At least four major factors apply crucial restraints to this line of *in vivo* research. First, the intentional tailoring of nanoscale features without the alteration of chemical properties remains crucial but difficult to achieve. Second, the translation of nanotopographical modifications to the surface of complex, three-dimensional architectures intended for precise-fit implantation is complicated. Third, the detection of cellular and molecular responses, which is inherent to *in vivo* material–cell interface research, requires access to cellular and molecular biomarkers immediately adjacent to or on the surface. Finally, and in our opinion the most critical factor for human experimentation, the intervention should enable detailed interfacial biomolecular interrogation while minimizing local tissue destruction, pain, and sequelae.

The objective of this study was to examine whether an ordered and well-characterized nanotopography superimposed on machined surfaces could promote a specific molecular signature for osseointegration in the human jaw bone. The implants had identical macro- and microscale configurations as well as identical surface chemistries, with the only variable being the presence or absence of a predetermined nanotopography pattern produced by colloidal lithography. Furthermore, quantitative polymerase chain reaction (qPCR) was used to investigate the molecular activities at the bone-implant interface.

## 2 Materials and Methods

### 2.1 Implants and Nanopatterning

Screw-shaped implants made of grade II titanium (Ti) (Elos Medtech Pinol A/S, Denmark) were used. The dimensions were 2 mm in diameter and 5 mm in length. Implant surface modifications were made to produce two subsets of implants, a control implant with a machined surface (M implant) and a test implant with a topographically nanopatterned machined surface (MN implant). Both types of implant were coated with a thin Ti film and heat-treated to provide identical surface chemistries. Nanopatterning was performed using colloidal lithography as described previously.^33^ In brief, negatively charged spherical polystyrene (PS) nanoparticles with a nominal diameter of 41 ± 6 nm (a 2% wt/wt colloidal solution of surfactant-free white polystyrene latex; Invitrogen Corp., Carlsbad, CA) were electrostatically adsorbed to the positively precharged surface of machined mini-implants. Precharging on the surfaces was achieved by immersion in 5% wt/wt aluminum chloride hydroxide polyelectrolyte (chlorohydrol, Summit Reheis, Huguenot, NY) for 30 s. The repulsive electrostatic interactions among the PS nanoparticles and attractive interaction toward the positively charged implant surface resulted in a short-range ordered pattern made of PS nanoparticles. The MN implants were then rinsed with Milli-Q water and dried in an oven at 103 °C for 2 min. Afterward, the residues of the polyelectrolyte were stripped off via oxygen plasma exposure at 150 W for 30 s (TePla 300PC, TePla AG, Germany). To achieve a homogeneous implant surface chemistry, a 30 nm thick Ti layer was sputter-coated (FHR MS150, FHR Anlagenbau GmbH) on both implant groups. Finally, all of the implants were heat-treated at 500 °C (high-temperature furnace, AWF 12/65, U.K.) for 5 h and kept in sterile glass vials until surgery.

### 2.2 Surface Characterization

Scanning electron microscopy (SEM, Zeiss Supra 40 VP, Carl Zeiss GmbH, Germany) was used to visualize the micro- and nanoscale surface topography of the mini-implants. Low-magnification images were taken using secondary electron mode (SE2 detector) and a large working distance (>10 mm) to ensure a long field of focus. Medium- and high-magnification images were recorded using an in-lens detector and a short working distance (<10 mm) to gain resolution. Topographical parameters of the nanopattern (the semispherical diameter, height, distribution density, surface coverage, and induced surface area) were measured or calculated based on previously described image analysis protocols and formulas.^33^ The microscale roughness of the implants was evaluated using optical profilometry (Wyko NT1100, Veeco Inc.) and Scanning Probe Image Processor (SPIP) version 5.1.3.0 software (Image Metrology A/S, Denmark). The surface chemistry of the prepared implants was assessed by energy-dispersive X-ray spectroscopy (EDS) and time-of-flight secondary ion mass spectroscopy (TOF-SIMS). EDS analysis was performed on the middle of the implant thread root (1 area per implant, *n* = 3 for each implant group) in the same scanning electron microscope using an X-Max 60 mm^2^ EDS detector (Oxford Instruments, U.K.), 8.5 mm working distance and 15 kV electron acceleration. TOF-SIMS analysis was performed on the flat end of the implant (2 spots per implant, *n* = 3 for each group) using the TOF-SIMS-5 instrument (IONTOF Technologies GmbH, Germany).

### 2.3 Surgical Procedure

The protocol was approved by the Regional Ethical Review Board in Gothenburg, Sweden (Dnr 620-16). Participants were recruited from the Maxillofacial Unit at the Sahlgrenska University Hospital in Gothenburg, Sweden. They were all referred to the clinic for implant placement in the posterior maxilla. Patients with good general health devoid of active oral pathologies (marginal or apical periodontitis) were included. Pregnancy, current smoking, uncontrolled metabolic disease, and previous radiotherapy to the head and neck served as exclusion criteria. Only patients with adequate bone volume, as judged radiographically (width > 4 mm, height > 6 mm), who were to receive implants in the posterior maxilla without the need of augmentation were included. Ten patients were included: four men and six women with an age range of 42–87 years and a mean age of 61.7 years (demographics presented in Table 1). All patients exhibited type 3 bone quality at the implant sites according to the Lekholm and Zarb classification.^45^ Informed written consent to participate in the study was obtained from each patient.

**Table 1:** Demographic Data from the Patients Included in the Study^a^

patient	age (years)	sex	medical background	medication	smoking	healing time (weeks)
1	87	male				7
2	57	female			ended 8 weeks prior to surgery	8
3	43	female				6
4	63	female	hypothyroidism	levaxin		6
5	42	male				8
6	56	female	hypercholesterolemia	atorvastatin		8
7	68	male	hypertension, hypercholesterolemia, myocardial infarction, hypothyroidism	trombyl, imdur, metoprolol, felodipin, losartan, simvastatin, levaxin		8
8	68	female	hypertension, urinary incontinence	losartan, betmiga		8
9	64	female				8
10	69	male				8

a Pregnant patients and patients with current smoking status, uncontrolled metabolic diseases, previous radiation therapy to the head and neck, or untreated oral diseases were excluded (*n* = 10).

Each patient received an MN and control (M) mini-implant at the same side of the maxilla. After local anesthesia, a mucoperiosteal flap was reflected, exposing the buccal and palatal recipient bone. After the clinical implant procedure had been completed, the experimental mini-implants were installed posterior to the clinical implants in the edentulous posterior maxilla. A single 2 mm diameter twist drill was used at 1500 rpm under sterile saline irrigation, reaching a depth of 5 mm.

Thereafter, the mini-implants were installed using a screwdriver, ensuring good primary stability. The wound was closed with nonresorbable polyamide 6 sutures (Ethilon 4-0, Ethicon). The procedure was completed under antibiotic coverage as a single prophylactic dose 1 h before surgery (2 g of amoxicillin or 600 mg of clindamycin perorally). Analgesics (4 g of paracetamol combined with 1600 mg of ibuprofen) were prescribed for peroral intake for 3–5 days. The wound was evaluated on postoperative days 10–14 for signs of infection, at which time the sutures were removed. At 6–8 weeks after implantation, the patients were recalled for installation of the healing abutments on the clinical implants. After local anesthesia and healing abutment installation, the mini-implants were carefully unscrewed using a manual screwdriver and placed in an RNA preservation medium (RNA Shield; Zymo Research, CA). The surgical site was thereafter approximated and closed with resorbable polyglactin sutures (Vicryl 4-0, Ethicon).

### 2.4 Quantitative PCR (qPCR)

After 6–8 weeks, the mini-implants were unscrewed using a manual hexagonal screwdriver (10 specimens/implant type, *n* = 10). The retrieval was performed with strict precautions taken to preserve the RNA. The implant-adherent cells were homogenized using RLT buffer with b-mercaptoethanol and a TissueLyser instrument (Qiagen GmbH, Hilden, Germany), followed by centrifugation at 16 000*g* for 3 min. Total RNA was then extracted from the separated aqueous phase using an RNeasy Micro Kit (Qiagen GmbH, Hilden, Germany). Reverse transcription (RT) of the total RNA was performed using a GrandScript cDNA synthesis kit (TATAA Biocenter AB, Gothenburg, Sweden). The cDNA was then stored at −20 °C until qPCR analysis. Predesigned validated primers targeting genes involved in major biological processes were purchased from TATAA Biocenter AB (Gothenburg, Sweden). The panel of genes included the inflammatory cytokines tumor necrosis factor-α (TNF-α), interleukin-6 (IL-6), monocyte chemoattractant protein-1 (MCP-1), and interleukin-10 (IL-10); the osteogenic differentiation and bone-related factors runt-related transcription factor 2 (RUNX2), alkaline phosphatase (ALP), osteocalcin (OC), and bone morphogenetic protein-2 (BMP-2); and the osteoclastic differentiation and bone remodeling and coupling factors calcitonin receptor (CTR), cathepsin K (CATK), receptor activator of nuclear factor-kappa B (RANK), receptor activator of nuclear factor-kappa B ligand (RANKL), and osteoprotegerin (OPG). Prior to targeted qPCR analysis, randomly selected samples from the two implant types were screened for a panel of 12 reference genes (TATAA Biocenter AB, Sweden) (Table 2). The expression stability of the reference genes was evaluated using geNorm^46^ and NormFinder^47^ software to determine the best reference gene(s) for normalization. According to geNorm and NormFinder, one reference gene was considered appropriate for normalization, and the reference gene with the most stable expression was PPIA; thus, was used for normalization. qPCR analysis was then performed to assess the 13 target genes and selected reference gene. Reaction volumes of 10 μL in duplicate were subjected to analysis on a CFX96 platform (Bio-Rad Laboratories, Inc., Hercules) with TATAA SYBR GrandMaster Mix (TATAA Biocenter AB, Sweden). Expression of the target genes was normalized to the expression of the selected reference gene. Normalized relative expression levels were calculated using the delta–delta *C*_q_ method and displayed a 90% PCR efficiency (*k* × 1.9^ΔΔcq^).^48^

**Table 2:** List of Human Reference Genes Used in Screening for the Most Stable Reference Gene(s) for Normalization

reference gene full name	abbreviation
18S rRNA	RRN18S
β-actin	ACTB
β-2-microglobulin	B2M
β-glucuronidase	GUSB
glyceraldehyde-3-phosphate dehydrogenase	GAPDH
hypoxanthine phosphoribosyltransferase 1	HPRT1
peptidylpropyl isomerase A^a^	PPIA^a^
60S acidic ribosomal protein P0	RPLP
TATAA-box binding protein	TBP
tubulin, β polypeptide	TUBB
tyrosine 3/tryptophan 5 -monooxygenase activation protein, ζ polypeptide	YWHAZ
ubiquitin C	UBC

a Peptidylpropyl isomerase A (PPIA) was selected as the best stable reference gene according to geNorm and NormFinder software.

### 2.5 Statistics

Before conducting the study, a statistical power analysis was carried out to determine the accurate number of patients who needed to be included in the study. The aim was to detect, differences in gene expression, if present, between cells adherent on the M versus MN implants. The G* Power tool was used (software version 3.1.9.2)^49^ based on previous comparable studies involving gene expression analysis in humans,^50^ which indicated that the required sample size per group was 10. The Wilcoxon signed-ranks test was applied to identify statistically significant differences in gene expression between the two implant types in the paired analysis. Furthermore, Spearman correlation analysis was conducted to assess differences in the expression of different genes and the collected patient demographic data (age, sex, current systemic illnesses, current medication use, and a healing time period of 6, 7, or 8 weeks after implantation). All statistical tests were conducted with IBM SPSS Statistics version 25. Differences between the two groups with a *P*-value of less than 0.05 were considered to be significant. The data presented in the graphs represent the mean and standard error of the mean (SEM).

## 3 Results

### 3.1 Surface Characterization

**Figure 1:**
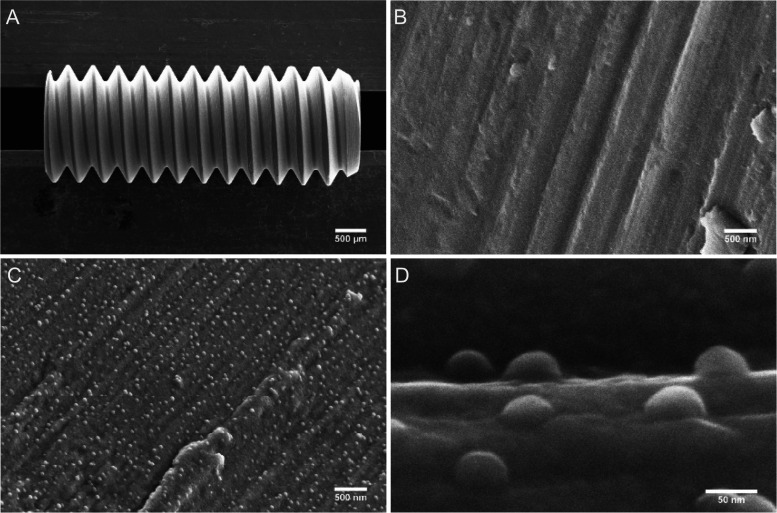
SEM evaluation: (A) Low-magnification overview of the mini-implant; (B) intermediate-magnification image of an implant with a machined surface; and (C) an implant with a nanopatterned surface. (D) High-magnification image of semispherical profiles on the nanopatterned surface. Images (B) and (C) were taken at the root of the implant thread. Image (D) was taken at the flank of the implant thread. All analyzed implants were sputter-coated with 30 nm titanium film and heat-treated.

Mini-implant geometry and surface topography at micro- and nanoscales are shown in Figure 1. Surface microgrooves created by tooling were seen in both implant groups (M and MN), but the MN group also contains a superimposed nanopattern consisting of semispheres of a uniform size (26 nm height, and 51 nm average diameter) with an ordered short-range distribution (see Table 3 for size and distribution parameters).

**Table 3:** Topographical Parameters of the Implants, as Analyzed by Optical Profilometry and SEM (*n* = 4)

roughness measurements of the two implant types		M	MN
microscale roughness parameters	mean roughness (Sa), nm	296 ± 19	261 ± 32
	root-mean-square roughness (Sq), nm	374 ± 20	325 ± 37
	skewness	–0.01 ± 0.06	0.08 ± 0.12
	kurtosis	3.1 ± 0.2	3.1 ± 0.2
	induced surface area, %	10.7 ± 0.3	10.2 ± 2.0
nanoscale pattern parameters	semisphere diameter, nm		51 ± 9
	semisphere height, nm		26 ± 4
	surface coverage, %		8 ± 3
	distribution density, μm^–2^		40 ± 5
	center-to-center distance, nm		130 ± 11
	induced surface area, %		9 ± 3

Further surface topography investigation by optical profilometry showed that nanopatterning did not affect microscale roughness (no significant difference in roughness parameters for *n* = 4 analyzed implants, Table 3). The measured kurtosis value is very close to 3, which indicates a normal roughness profile distribution without extreme extrusions or intrusions.

A degree of skewness close to zero also confirmed that peaks and valleys did not predominate the roughness profile. Based on the roughness amplitude level (an arithmetical mean height (Sa) and a root-mean-square height (Sq) less than 1 μm), the implants were categorized as having smooth surfaces.^51^

**Figure 2:**
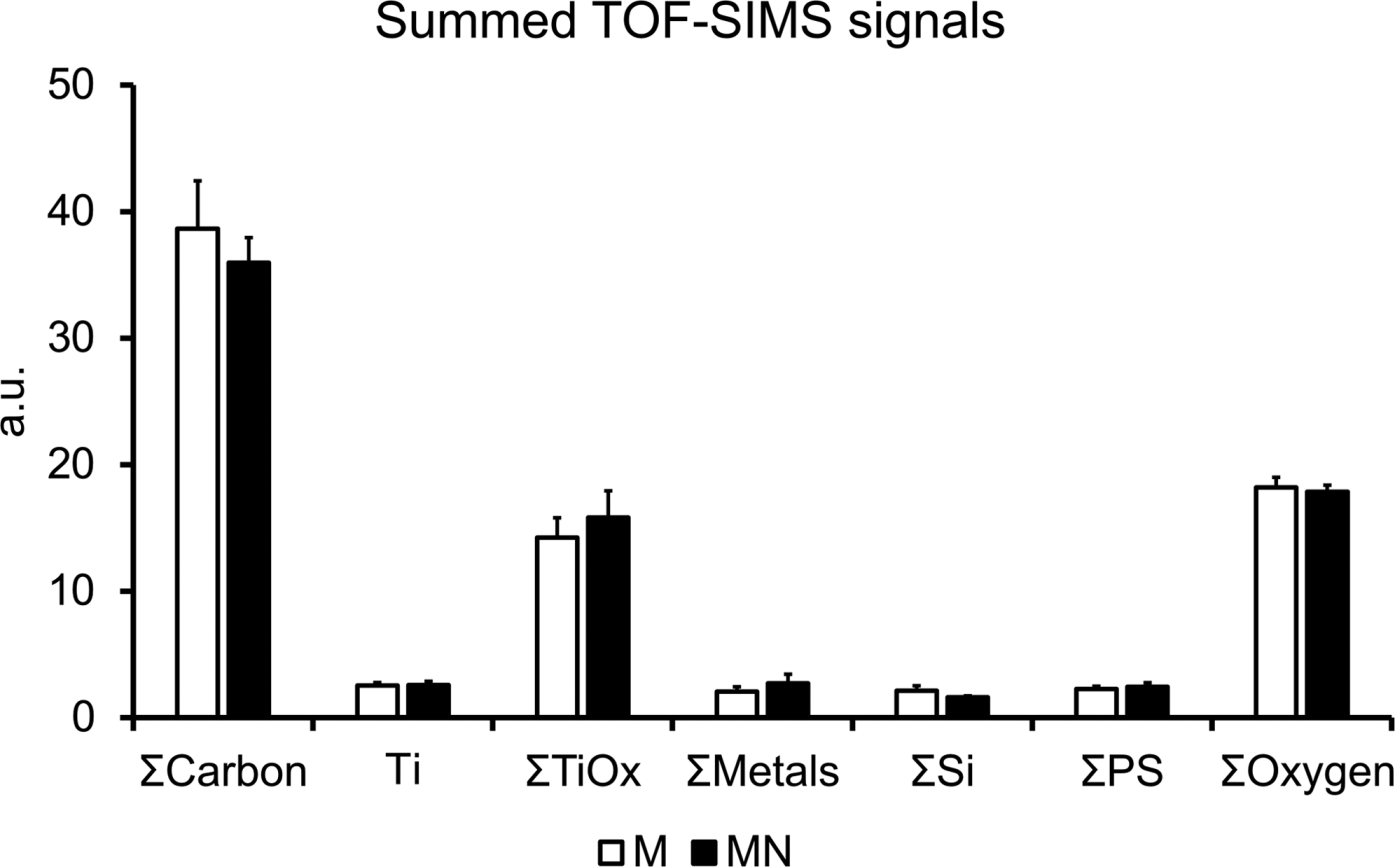
Chemical analysis of implant surfaces using time-of-flight secondary ion mass spectrometry (TOF-SIMS, *n* = 3). The normalized intensities of the following ions were summed and are shown by the plotted bars: ∑C = CH_3_^+^, C_2_H_5_^+^, CH_4_N^+^, C_3_H_5_^+^, C_3_H_7_^+^, C_4_H_7_^+^, C_6_H_5_^+^, C_7_H_7_^+^, C_9_H_7_^+^, C_14_H_30_NO_2_^+^, C__2_H^–^, CN^–^, C__16_H___31O__2_^–^, C__18_H__35_O__2_^–^; ∑TiO_x_ = ^∧^47TiO^+^, TiO^+^, TiO_2_H^+^, Ti_2_O_3_^+^, Ti_2_O_4_H^+^, Ti_3_O_5_^+^, Ti_4_O_6_^+^, Ti_4_O_7_^+^, Ti_6_O11^+^, TiO__2_^–^, TiO__3_^–^, TiO__3_H^–^, Ti__2_O__5_H^–^, Ti__3_O__7_H^–^, ∑Metals = Na^+^, Al^+^, K^+^, Ca^+^, V^+^, Fe^+^, CaOH^+^, Ni^+^, Cu^+^, FeOH^+^, Pb^+^; ∑Si = SiO__2_^–^, SiHO__3_^–^, Si__2_HO__5_^–^, and Si__3_HO__7_^–^; ∑PS = positive ions with masses 103, 128, 152, 165, and 178 u; negative ions with masses 37, 49, 62, and 73 u; ∑Oxygen = O^–^, OH^–^.

EDS analysis (Table 4) showed the presence of Ti, O, and C atoms on the surfaces of the implants, with traces of Al and Si. The presence of C (4–6%) can be attributed to atmospheric hydrocarbons adsorption to the surface. The Si (<0.1%) could have originated from the glass vials used for implant storage. The Al (0.1%) was likely part of the bulk Ti. The detected Ti/O atomic concentration ratios exceeded the stoichiometric ratio of titanium dioxide because metallic Ti dominated the probing spot volume, as the X-ray signal came from a depth of up to 1 μm. Heat treatment caused the O concentration to increase almost 4-fold and significantly reduced the Ti signal, indicating that part of the previously metallic Ti has formed oxides on the surface. The analysis confirmed that the chemical compositions of the M and MN implant groups did not significantly differ. This finding was supported by TOF-SIMS, in which not only could Ti, O, and C ions be investigated but also ion species comprising the specific fingerprints of polystyrene molecules in the M and MN implant groups could be compared (Figure 2).

**Table 4:** Chemical Analysis of Implants by EDS (Atomic Concentration in %, *n* = 3)

implant type	Ti (%)	O (%)	C (%)	Al (%)	Si (%)
before Ti-coating and heat treatment	81.9 ± 0.4	12.3 ± 0.2	5.7 ± 0.2	0.13 ± 0.02	0.09 ± 0.03
M	55.7 ± 1.6	40.1 ± 1.6	4.1 ± 0.2	0.06 ± 0.01	0.03 ± 0.01
MN	53.9 ± 0.6	41.7 ± 0.7	4.3 ± 0.2	0.08 ± 0.01	0.04 ± 0.003

### 3.2 Quantitative Polymerase Chain Reaction (qPCR)

**Figure 3:**
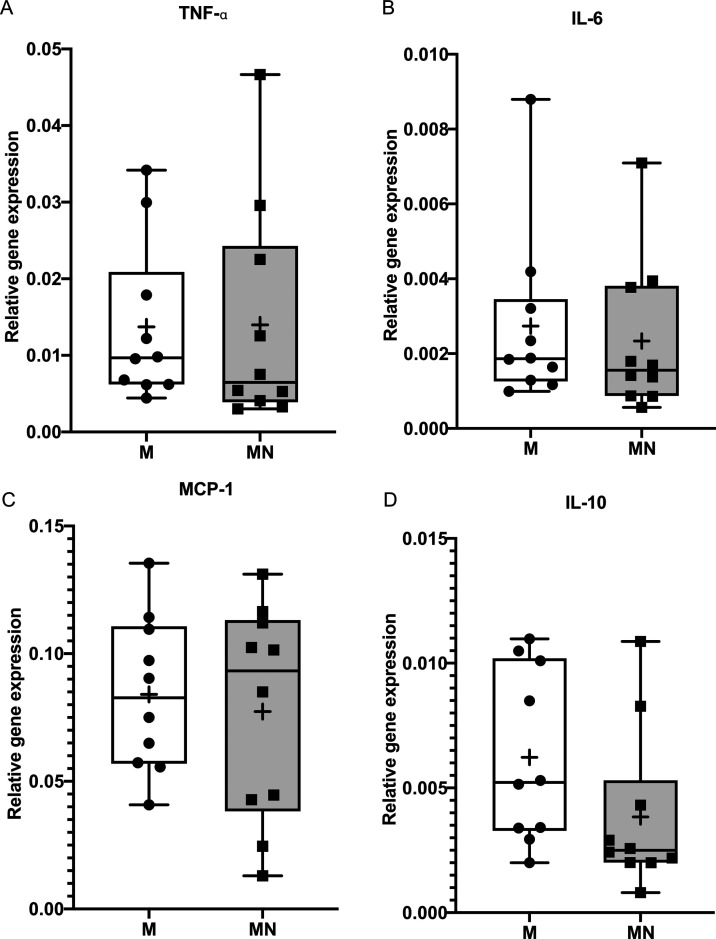
Boxplots showing the gene expression of inflammatory cytokines and chemokines in cells adherent to implants with a machined surface (M implants) and implants with a machined surface with superimposed nanotopography (MN implants) at retrieval after 6–8 weeks. (A) TNF-α, (B) IL-6, (C) MCP-1, and (D) IL-10. The data show the mean and standard error of the mean (*n* = 10). The boxplots show the median (line), mean (plus), first and third quartiles (box), minimum and maximum (whiskers), and all data values for the individual patients.

**Figure 4:**
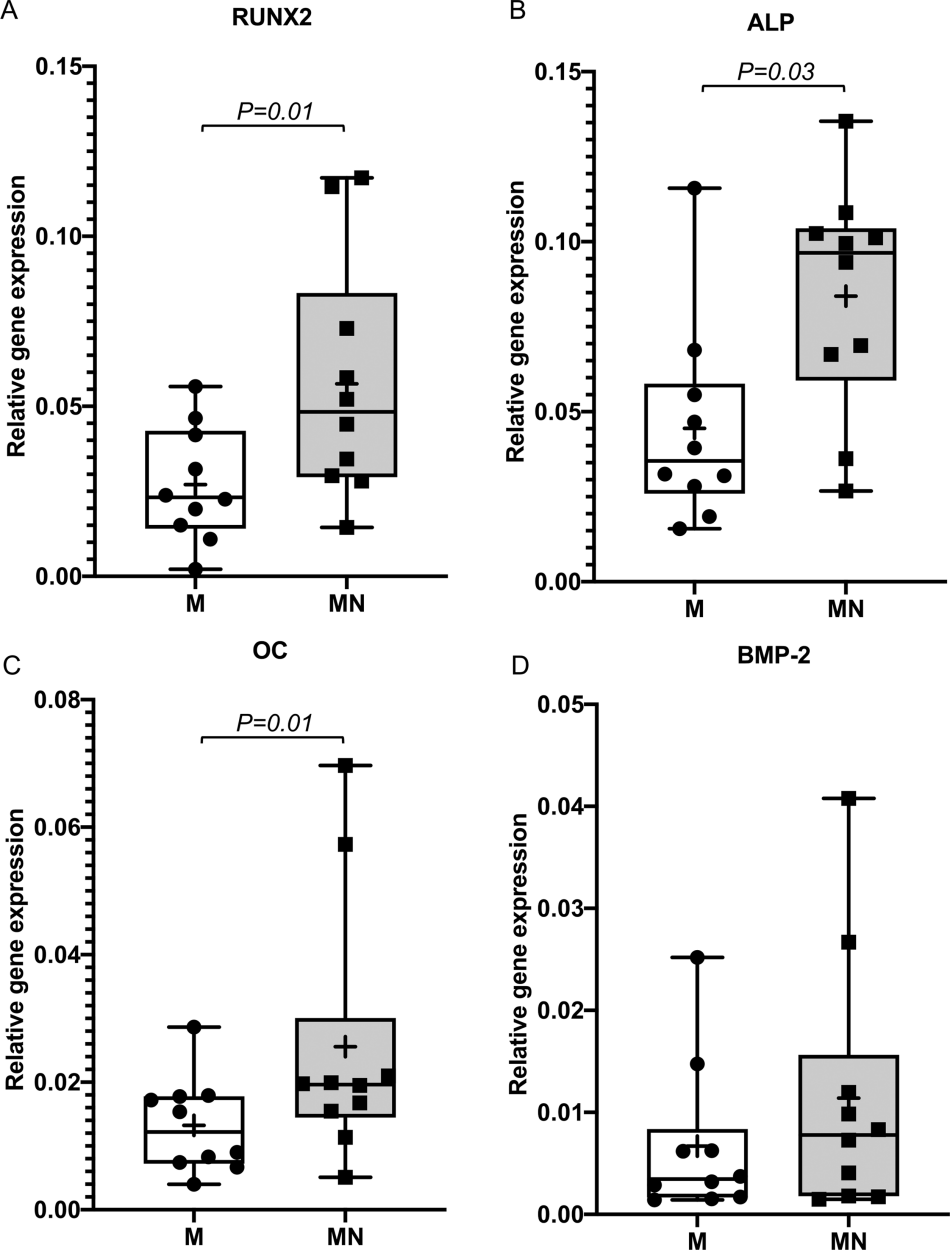
Boxplots showing the expression of genes related to osteogenic activity and osteoblastic differentiation in cells adherent to implants with a machined surface (M implants) and implants with a machined surface with superimposed nanotopography (MN implants) at retrieval after 6–8 weeks. (A) RUNX2, (B) ALP, (C) OC, and (D) BMP-2. The data show the mean and standard error of the mean (*n* = 10). Significant differences are indicated (*p* < 0.05). The boxplots show the median (line), mean (plus), first and third quartiles (box), minimum and maximum (whiskers), and all data values for the individual patients.

**Figure 5:**
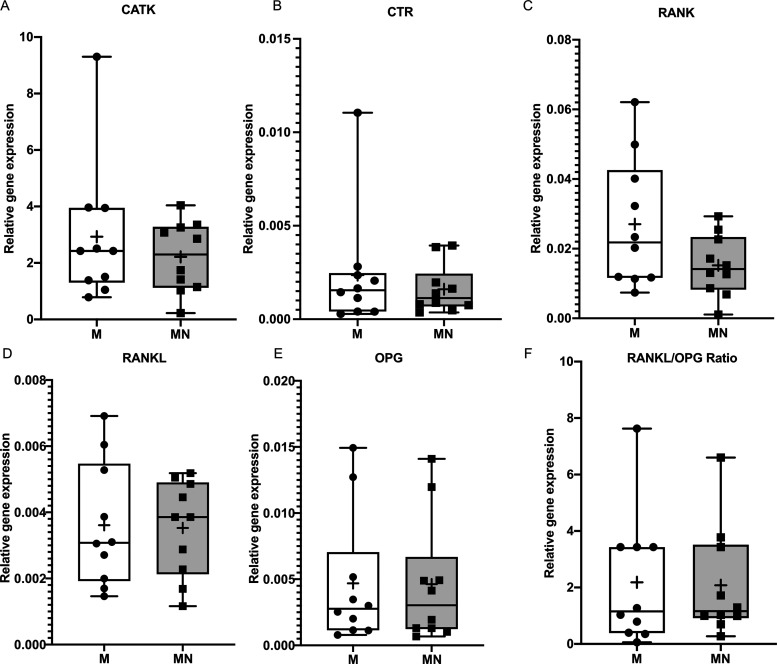
Boxplots showing the expression of genes related to osteoclastic and osteoblast–osteoclast coupling activity in cells adherent to implants with a machined surface (M implants) and implants with a machined surface with superimposed nanotopography (MN implants) at retrieval after 6–8 weeks. (A) CTR, (B) CatK, (C) RANK, (D) RANKL, (E) OPG, and (F) RANKL/OPG ratio. The data show the mean and standard error of the mean (*n* = 10). The boxplots show the median (line), mean (plus), first and third quartiles (box), minimum and maximum (whiskers), and all data values.

The expression of selected genes by implant-adherent cells is presented in Figures 3–5. The data are divided into three groups representing major biological responses: (1) inflammation; (2) osteogenic differentiation and bone formation; and (3) osteoclastic differentiation, remodeling, and coupling.

#### 3.2.1 Gene Expression of Inflammatory Cytokines

Expression levels of the proinflammatory cytokines TNF-α and IL-6 and the chemokine MCP-1 were similar in cells adherent to the M and MN implants. Similarly, expression of the anti-inflammatory cytokine IL-10 did not significantly differ between cells adherent to the two implant types (Figure 3).

#### 3.2.2 Gene Expression of Factors Related to Osteogenic Differentiation and Bone Formation

Expression of the osteogenic commitment transcription factor RUNX2 was significantly higher (by 2-fold) in cells adherent to the MN implant versus those adhered to the M implant (Figure 4A). Similarly, expression of the osteogenic differentiation and bone formation markers ALP and OC, respectively, was significantly higher (by 1.7- and 2-fold) in cells adherent to the MN implants versus those adherent to the M implants (Figure 4B,C). The 1.7-fold increase in BMP-2 expression in cells adherent to the MN implant versus those adherent to the M implant did not reach statistical significance (Figure 4D).

#### 3.2.3 Gene Expression of Factors Related to Osteoclastic Differentiation and Bone Remodeling Coupling

Although the expression of osteoclastic genes (CATK, CTR, and RANK) tended to be decreased by 1.4- to 1.7-fold in cells adherent to the MN implants versus M implants, these differences were not statistically significant (Figure 5A–C). The expressions of genes related to bone remodeling coupling (RANKL and OPG) and RANKL/OPG ratio were similar in cells adherent to the two implant types (Figure 5D–F).

### 3.3 Correlation Analysis

Increased age was associated with increased expression of the proinflammatory cytokine TNF-α and chemokine MCP-1 in cells adherent to the M implant and reduced expression of the pro-osteogenic growth factor BMP-2 in cells adherent to the MN implant (Table 5). In addition, older patient age showed a positive correlation with the expression of the anti-inflammatory cytokine IL-10 in cells adherent to the MN implant. Female sex was positively associated with the expression of the osteogenic commitment gene RUNX2 and negatively associated with the osteoclastic gene RANK in cells at the M implants. In contrast, female sex showed positive associations with the expression of the proinflammatory chemokine MCP-1 and the anabolic coupling gene OPG when the MN implants were analyzed. Further, although the M implants showed positive associations between hypertension and the angiotensin II-converting enzyme drug administration with increased expression of the proinflammatory chemokine MCP-1, the MN implants showed positive associations between hypercholesterolemia and statin drug administration with reduced expression of proinflammatory IL-6. Further, the expression of IL-6 in cells at the MN implant was negatively associated with the occurrence of several illnesses.

**Table 5:** Correlation Analysis^a^

	M		MN	
	positive correlations r (*P*-value)	negative correlations r (*P*-value)	positive correlations r (*P*-value)	negative correlations r (*P*-value)
older age	TNF-α 0.7 (0.02)		IL-10 0.7 (0.3)	BMP-2 -0.7 (0.03)
	MCP-1 0.8 (0.001)			
female sex	RUNX2 0.8 (0.002)	RANK -0.7 (0.02)	MCP-1 0.7 (0.02)	
			OPG 0.8 (0.008)	
hypertension	MCP-1 0.7 (0.02)			
angiotensin R blocker	MCP-1 0.7 (0.02)			
hypercholesterolemia				IL-6 -0.7 (0.02)
several illnesses				IL-6 -0.7 (0.03)
statin drugs				IL-6 -0.7 (0.02)
several medications				IL-6 -0.7 (0.03)

a Data show positive and negative correlations between the patients’ demographic data/medical conditions and gene expression in cells adherent to implants with a machined surface (M) or implants with a machined surface and superimposed nanopattern (MN) (*n* = 10).

## 4 Discussion

This study demonstrates that a predetermined nanotopography with a uniform size (51 ± 9 nm in diameter and 26 ± 4 nm in height), shape (hemispherical protrusions), and distribution density (40 ± 5 μm^–2^) modulated osteogenic differentiation and the expression of bone formation-related genes in the human jaw bone. These novel findings in humans verify and extend previous findings in an experimental animal model in which identical MN implants and a similar sampling approach were used to analyze implant-adherent cells.^33,44^ In the experimental animal study, the MN implants stimulated increased expression of the osteogenic differentiation markers ALP and OC compared to their expression in cells adhered to the M implants, which were devoid of a nanopattern, at 3 days after implantation in a rat tibia model. Further, the MN implant with controlled nanotopography superimposed on a microrough machined surface promoted implant stability during the development of osseointegration.^44^ Considering the temporal regenerative differences between small animal models and humans, collectively, the data reveal the pro-osteogenic potential of nanotopography alone during the early stage of healing after Ti implantation.

The transcription factor RUNX2 was upregulated at MN implants compared to the M implants. RUNX2 is a key transcription factor in the commitment of MSCs to differentiation along the osteoblastic lineage and not other lineages.^52^ Additionally, the increase in RUNX2 expression was observed in parallel with upregulation of the bone-specific genes OC and ALP.^53,54^ Studies on the influence of implant surface properties on molecular activities at the bone-implant interface in humans are sparse, especially with respect to the role of nanoscale topography. Bryington and co-workers compared sandblasted (microrough) surfaces with and without hydrofluoric acid treatment, a method proposed to develop nanoscale roughness on the implant surface.^50^ Although SEM confirmed the development of nanofeatures superimposed on the microirregularities, neither the shapes, sizes nor distributions of these features appeared consistent.^50^ Furthermore, the acid etching process is likely to influence surface chemistry and oxide properties, which may exert confounding effects of the role of nanoscale topography. Nonetheless, through the use of genome-wide microarray analysis, the study showed that OC and the other osteogenic transcription factor osterix (OSX) were upregulated in cells adherent to implants with acid-induced nanofeatures after 7 days of implantation.^50^ Taken together, the findings of these studies suggest that the pro-osteogenic effect of nanoscale topography is mediated on the transcriptional level via the upregulation of factors that drive the differentiation of MSCs toward the osteogenic linage.

Attempts have been made to explore the *in vivo* effects of controlled nanopatterns on the biological processes of osseointegration in animal models. One such example is the application of controlled anodization processing, using low voltages, to produce nanotopographies on different scales in the form of nanotubes.^28,39,55^ Using this approach, Wang et al. explored the effects of 30, 70, and 100 nm nanopatterns produced by the anodization of machined cylindrical Ti implants using 10, 20, and 30 V, respectively, in an 0.3% NH_4_HF_2_ electrolyte solution.^28^ After multiple periods (1, 2, 3, 4, and 5 weeks) of healing in minipig calvaria, the expression levels of all bone-related genes tested (ALP, OSX, and Collagen (COLL)) were upregulated in tissue adherent to the MN versus M implants, with the highest relative expression observed for 70 nm nanotubes.^28^ In line with these findings, increased BIC was observed for all MN implants, with the highest percentage reported for the 70 nm MN implants. Based on these findings, it can be assumed that there is a synergistic bone-promoting effect induced by the combination of ordered microroughness (machined microgrooves) and the ordered nanopattern. However, such a synergistic effect has also been observed for irregular microroughness when it was patterned with an ordered nanoscale topography.^56^ In the latter animal study, irregular microroughness, produced by sandblasting/acid etching, promoted the highest BIC when patterned with 80 nm nanotubular topography, in contrast to 50 nm, 30 nm, and unpatterned.^56^ Moreover, another combination of irregular microscale roughness with ordered nanoscale pores, produced by anodic oxidation using sodium tetraborate electrolyte, promoted the highest bone formation compared to microrough implants without the nanopores.^57^ Taken together, the present human and previous animal *in vivo* data suggest that the synergistic bone-promoting effect of the combination of micron- and nanoscale roughness is largely dependent on the type of the nanoscale topography (*e.g.*, size, shape, and/or density) rather than the specific shape of the underlying microscale roughness, whether it is ordered or irregular. This assumption is at least partly supported by a recent study comparing two nanopatterns superimposed on microrough implants produced by selective laser melting.^58^ In that study, the ordered 70 nm nanotubular pattern, fabricated by anodic oxidization, resulted in less osteoclastic activity, *in vitro* and *in vivo*, higher bone formation activity, *in vitro* and *in vivo*, and higher BIC *in vivo*, compared to an irregular 100–120 nm nanotopography produced by alkali heat treatment on similar microrough implants.^58^ Collectively, these findings from *in vivo* studies, even if limited in number at present, indicate an enhanced osseointegration in response to ordered nanopattern superimposed on a microroughness. These *in vivo* findings are further supported by several *in vitro* studies showing that such a combination of ordered nanopatterns and microroughness promote higher adhesion of MSCs and osteoblasts, osteoblastic differentiation, osteogenic activity, and mineral deposition.^31,59−61^

The specific effects of nanotopography on the molecular activities of bone in contact with the implant surface and the structural development and adaptation of this bone have been addressed in an animal model.^44^ Therein, the application of a statistical interaction model suggested that although BIC was predominantly influenced by underlying microscale topography, the significant contribution of nanotopography based on the degree of implant stability and the regulation of inflammatory gene expression in the implant-adherent cells was evident.^44^

These findings may provide a partial explanation for the observed profound effects of the nanotube topographies during multiple periods of osseointegration,^28^ in contrast to the bone-promoting effect of the present nanotopography model, which was mainly observed during the first week after implantation.^33^ Therefore, the extended upregulation of bone-related genes in parallel with a progressively increasing BIC may be due to the combined effects of nanoscale topography and other surface properties, including microtopography, surface chemistry, and oxides, which are altered by the anodic oxidization process. This assumption is further supported by other studies showing that anodically oxidized implants induced the extended upregulation of bone-related gene expression over several stages of osseointegration in parallel with a progressive increase in BIC.^15,16,62^

Interestingly, the surfaces resulting from the two methods of anodic oxidation (i.e., low voltage^28^ and high voltage^15,16,62^) also triggered a significant increase in the expression of bone remodeling-related genes (TRAP and CATK) in the implant-adherent cells, an observation that was not evident when the effect of controlled nanotopography alone was evaluated in the present human experiments and previous animal experiments.^32,33,44^

In the present study, no difference in the expression of inflammatory cytokines was detected between the MN versus M implants. This finding is in contrast with the results from an animal model in which similar MN implants significantly downregulated the expression of major proinflammatory cytokines (TNF-α and MCP-1 in the implant-adherent cells).^32,33^ One possible reason for this difference is that the analysis in the present study was performed at a relatively late time point, at which point the acute inflammatory response triggered by surgery and implantation had resolved, precluding a comparison with early differences in inflammation between the two surfaces. This assumption is partly supported by the observation that at 3 days after implantation, the acid-induced nanotopography significantly upregulated the expression of immunomodulatory cytokines (IL-9, IL-22, and TOLLIP),^50^ all of which have been implicated in downregulation of the inflammatory response.^63−65^

Correlation analysis suggested that patient-related factors, such as age and sex, may have different impacts on implant-adherent gene expression depending on the implant surface. Here, we found an increased age to be associated with increased proinflammatory activity in cells adherent to M implants as well as a reduction in the regenerative potential of the cells adherent to the MN implants.

A precise explanation for the correlation findings cannot be provided at the moment. However, a machined surface has been reported to be associated with the upregulated expression of proinflammatory cytokines.^16,32,33,62,66^ Although speculative, such machined surface-induced upregulation may be further augmented with increased recipient age. On the other hand, assuming that surface-modified MN implants exert an anti-inflammatory effect, this effect may have mitigated the age-related upregulation of TNF-α and MCP-1. The latter assumption is supported by the observation that increased age was associated with upregulation of the anti-inflammatory cytokine IL-10 in cells adherent to the MN implants. The interesting observations regarding differential gene response in cells adherent to the MN implants with respect to age, sex, the occurrence of systemic illnesses, and medication use warrant further clinical studies to investigate the impact of each of these factors on the molecular mechanisms of osseointegration.

A limitation of this study was that due to ethical and practical reasons, no attempt to include biomechanical data was made. Another limitation is that the study included neither gene expression analysis nor histological evaluation of peri-implant bone. On the other hand, the advantages of analyzing gene expression in the implant-adherent cells provided a major strength. Implant-adherent cells have been shown in many studies in animal models and humans to “sense” implant surface properties and to act as an indicator of surface-regulated molecular activities in osseointegration, in contrast to cells in the peri-implant bone.^16,32,33,62,66^ This postulation is supported by findings from a systematic review of studies that involved genomic analyses of osseointegration in humans.^67^ Although global gene expression using microarray techniques provided a general view on biological processes involved during different phases of osseointegration,^68−70^ specific regulations of osteogenesis and bone-related genes in response to different implant surfaces were mainly detected in implant-adherent cells, using quantitative real-time PCR technique.^50^ In addition, the nondestructive implantation and subsequent unscrewing of the mini-implants served as a relatively noninvasive yet reliable approach to investigate molecular activities in the interface of human bone with the Ti implant.

## 5 Conclusions

Based on the results of the present study, it can be concluded that an intentional, controlled nanopattern in the form of hemispherical 51 ± 9 nm protrusions promoted the expression of genes related to early osteogenic differentiation and osteoblastic activity in implant-adherent cells in human bone. In addition, correlation analysis suggested that nanopatterns may mitigate an age-related increase in proinflammatory activity in implant-adherent cells.

## Notes

All study data are included in the manuscript.

## ABBREVIATIONS

TNF-α: tumor necrosis factor-α
IL-6: interleukin-6
MCP-1: monocyte chemoattractant protein-1
IL-10: interleukin-10
RUNX2: runt-related transcription factor 2
ALP: alkaline phosphatase
OC: osteocalcin
BMP-2: bone morphogenetic protein-2
CTR: calcitonin receptor
CATK: cathepsin K
RANK: receptor activator of nuclear factor-kappa B
RANKL: receptor activator of nuclear factor-kappa B ligand
OPG: osteoprotegerin
PPIA: peptidylprolyl isomerase A
